# Characterization of the biosynthesized intracellular and extracellular plasmonic silver nanoparticles using *Bacillus cereus* and their catalytic reduction of methylene blue

**DOI:** 10.1038/s41598-022-16029-1

**Published:** 2022-07-21

**Authors:** Nada Alfryyan, Mohamed G. M. Kordy, Mohammed Abdel-Gabbar, Hanan A. Soliman, Mohamed Shaban

**Affiliations:** 1 Department of Physics, College of Sciences, Princess Nourah Bint Abdulrahman University, P.O. Box 84428, Riyadh, 11671 Saudi Arabia; 2 Biochemistry Department, Faculty of Science, Beni-Suef University, P.O. Box 62521, Beni-Suef, Egypt; 3 Nanophotonics and Applications (NPA) Lab, Physics Department, Faculty of Science, Beni-Suef University, Beni-Suef, 62514 Egypt; 4 Department of Physics, Faculty of Science, Islamic University of Madinah, P.O. Box: 170, Al-Madinah Al-Munawarah, 42351 Saudi Arabia

**Keywords:** Biological techniques, Biotechnology, Microbiology, Environmental sciences, Chemistry, Materials science, Nanoscience and technology

## Abstract

The biosynthesis of silver nanoparticles (Ag NPs) has been studied in detail using two different approaches. For the first time, *Bacillus cereus* is used for one-pot biosynthesis of capsulated Ag NPs, using both intracellular and extracellular approaches. To discriminate between the produced nanostructures by these two approaches, their structures, nanomorphologies, optical properties, hydrodynamic sizes and zeta potentials are studied using different techniques. Fourier-transform infrared spectroscopy was used to identify the bioactive components responsible for the reduction of Ag^+^ ions into Ag and the growth of stable Ag NPs. Scanning and transmission electron microscopy images displayed spherical and polygon nanomorphology for the intracellular and extracellular biosynthesized Ag NPs. For intracellular and extracellular biosynthesized Ag NPs, a face-centred cubic structure was observed, with average crystallite sizes of 45.4 and 90.8 nm, respectively. In comparison to the noncatalytic reduction test, the catalytic activities of intracellular and extracellular biosynthesized Ag NPs were explored for the reduction of highly concentrated MB dye solution. Extracellular Ag NPs achieved 100% MB reduction efficacy after around 80 min, compared to 50.6% and 24.1% in the presence and absence of intracellular Ag NPs, respectively. The rate of MB reduction was boosted by 22 times with the extracellular catalyst, and by 3 times with the intracellular catalyst. Therefore, the extracellular production process of Ag NPs utilizing *Bacillus cereus* bacteria might be applied in the industry as a cost-effective way for eliminating the toxic MB dye.

## Introduction

Nanomaterials are particles with a size of 1 to 100 nm with unique physicochemical and biological properties^1^. These nanostructured materials have emerged as a robust platform for a number of applications due to their unique physicochemical and biological features at the nanoscale^2–5^. Several methods for manufacturing nanomaterials are now being investigated in an attempt to provide solutions to many of humanity's current concerns^6–10^. Physical, chemical, and biological methods are all used to synthesize nanoparticles^11^. Chemical and physical procedures are quite costly, as they require toxic chemicals and high energy consumption^12^. Aside from that, the synthesised nanoparticles are not as pure as predicted, and their surfaces can be sedimented by chemicals. Furthermore, preparing nanoparticles involves an additional processing step to prevent particle aggregation which makes them highly difficult. In comparison to chemical and physical methods, the biological synthesis or biosynthesis of nanoparticles by microorganisms or plant extracts results in a more controllable size and shape of the nanoparticles and does not require the use of toxic and harmful substances^13,14^. Biosynthesis can be accomplished utilizing different plants, bacteria, fungi, yeast, and actinomycetes^8,15–18^. Therefore, the biosynthesis of nanoparticles contributes to the sustainable development goals, while the alternative physical and chemical methods exclusively utilize scarce and expensive resources for nanoparticle synthesis^19–22^. Also, biosynthesis typically happens at a relatively ambient temperature and pressure, as documented in numerous articles, making this approach more preferred than other methods^20,23–30^. Although the benefits of biosynthesis of nanoparticles, recent reports indicate that this approach still needs more research and efforts to get more uniform nanoparticles in shape and size and to avoid the agglomeration^31,32^. Also, for the seek of commercialization, low-cost and massive product bio extracts and microorganisms must be used.

Among the biosynthesis nanoparticles, silver (Ag) nanoparticles received a lot of attention because of their localized surface plasmon resonance (LSPR), physicochemical properties, catalytic activity and their wide range of applications^31–33^. The hydrophilicity, stability, and large surface area of the biosynthesized AgNPs are preferable for different applications^34,35^. Bamal et al. reported the preparation of Ag NPs utilising biological methods via plant extract^36^. When compared to chemical synthesis techniques, microorganisms-based synthesis is more cost-effective, simple, reproducible, and uses less energy. Klaus et al. reported for the first time the manufacturing of single nanocrystals AgNPs with well-defined compositions utilizing *Pseudomonas stutzeri* bacteria, which is known as silver mine bacteria^37^. Recently, Al-Rajhi et al. reported the biosynthesis of Ag NPs with less toxic by-product generation, greater stability, and lower toxicity to healthy cells^12^. The photoinduced biological approach, photo-biosynthesis approach, that used to produce Ag NPs, is not completely understood in the earlier studies and acquired extensive research to provide the concept of this method and the possible mechanistic mode of synthesis as updated by Desai et al.^38^. Three stages are involved in the mechanism of bacterial biosynthesis of Ag NPs and other metallic nanoparticles. Firstly, the Ag atoms are formed through the bio-reduction of Ag^+^ ions which are bio-catalyzed by enzymes like nitrate reductase^39^. The nitrate reductase enzyme, in bacteria, reduces the nitrate anion into nitrite anion and then converts nitrite anion into ammonia^40^. I.e., the cation is reduced into metal through the nitrate reductase enzyme which can be present in the utilized bacteria. Secondly, the Ag atoms that are produced after the reduction of Ag^+^ ions nucleate to develop silver nanoparticles. Thirdly, the formed Ag nanoparticles are stabilized by encapsulating the formed Ag nanoparticles with bioactive components or linking the surface of the nanoparticles with excessive negatively charged reducing agent ions.

Microorganisms' generation of Ag NPs can be classified as intracellular or extracellular biosynthesis. Extracellular synthesis is easier and more straightforward than intracellular synthesis because the nanoparticles produced may be easily purified and retrieved^41,42^. Intracellular production, on the other hand, demands centrifugation followed by a series of ultrasonic cycles to break the cells, making the purification step more challenging. Intracellular synthesis of nanoparticles includes the transportation of ions and molecules into the bacterial cells in the presence of enzymes^43,44^. As a result, the intracellular process may take a longer time to create nanoparticles than the extracellular mechanism, as reported by Gemeel Abd et al.^24^. *Kocuria rhizophila*, *Bacillus subtilis*, *Acinetobacter calcoaceticus*, *Bacillus amyloliquefaciens*, *Bacillus flexus*, *Bacillus megaterium*, *Streptomyces coelicolor*, *Lactobacillus rhamnosus*, *Ochrobactrum rhizosphaerae*, *Nostoc commune*, and* Staphylococcus aureus* have all been employed for both intracellular and extracellular production of Ag^1,45^. Gemeel Abd et al.reported that he biosynthesized Ag NPs by the intracellular method utilizing *Pseudomonas aeruginosa* also had antibacterial activity against the bacterium that was employed in the creation of these nanoparticles^24^. So, *Pseudomonas aeruginosa* bacteria establish a defence or resistance mechanism to protect themselves from the toxic salt of Ag^+^ ions by converting them to Ag NPs, which are later used to kill them^46^. Therefore, it is important to explore the application of widely spread microorganisms such as *Bacillus cereus *(*B. cereus*) for the biosynthesis of Ag nanoparticles with controlled shapes (spheres, rod triangles, wires, hexagons, cubes, and pentagons) and sizes by the intracellular and extracellular technique. *B. cereus* is a gram-positive, rod-shaped, facultatively anaerobic, motile, beta-haemolytic, and spore-forming bacteria found in soil, food, and marine sponges. *B. cereus* can metabolize a variety of compounds including carbohydrates, proteins, peptides, and amino acids for growth and energy^47^. Shue et al. stated that the bio-molecules of reductive amino acids, α-linolenic acid, and carbohydrates in the yeast extract have a significant role in reducing Ag^+^ to Ag NPs utilizing NADH-dependent reductase or the nitrate reductase enzymes^17^. Electrostatic stabilizing agents can also govern the size of stabilized AgNPs through the capping process in intracellular or extracellular biosynthesis, which requires bio-stabilizers to be adsorbed onto the surface of the generated AgNPs^21^. *B. cereus* may produce metabolites, surfactants, bacteriocins, enzymes, and toxins into the biofilm, all of which can affect the biofilm and/or its environment^44,45^. Therefore, we expect that these components can contribute to the low-cost and widespread intracellular or extracellular manufacturing of controlled AgNPs, which can help in the control of size and shape, by reducing Ag^+^ ions and encapsulating the created Ag NPs. Also, it is important to explore and compare the catalytic performance of the prepared nanoparticles using the two different techniques. Singh and Dhaliwal developed a grafted copolymer of polyguar gum/acrylic acid with Ag NPs as a new adsorbent nanocomposite for removing MB dye from aqueous solutions^48^. Kordy et al. recently reported the biosynthesis of green coffee capped-Ag NPs with a face-centred cube and spherical shape with an average diameter of 8.6 nm using an aqueous green coffee extract^46^. These capped Ag NPs showed exceptional catalytic activity of 96% in 12 min for the degradation of 50 ppm methylene blue (MB) dye, as well as good antioxidant properties.

Here, to distinguish between the generated nanostructures, for the first time, *B. cereus* is employed for one-pot biosynthesis of capsulated Ag NPs, employing both intracellular and extracellular techniques. Various methods such as UV/Vis spectrophotometer, XRD (X-Ray Diffraction), FTIR (Fourier Transform Infra-Red), SEM (Scanning Electron Microscope), TEM (Transmission Electron Microscope), DLS (Dynamic Light Scattering) size, and zeta potential are used to explore the differences in the structures, morphologies, sizes, surface charges, function groups, and optical absorbance of the manufactured samples. In the presence of NaBH_4_, the samples were used to catalyse the reduction of the MB dye. In terms of kinetic rate constant and catalytic activity, the performance of intracellular and extracellular Ag NPs is compared to that of the noncatalytic process. Finally, in addition to the catalytic activity of the biosynthesized Ag NPs samples, this paper reveals the intracellular and extracellular mechanisms of Ag NP biosynthesis.

## Results and discussion

### Optical absorbance of intracellular and extracellular Ag NPs

**Figure 1:**
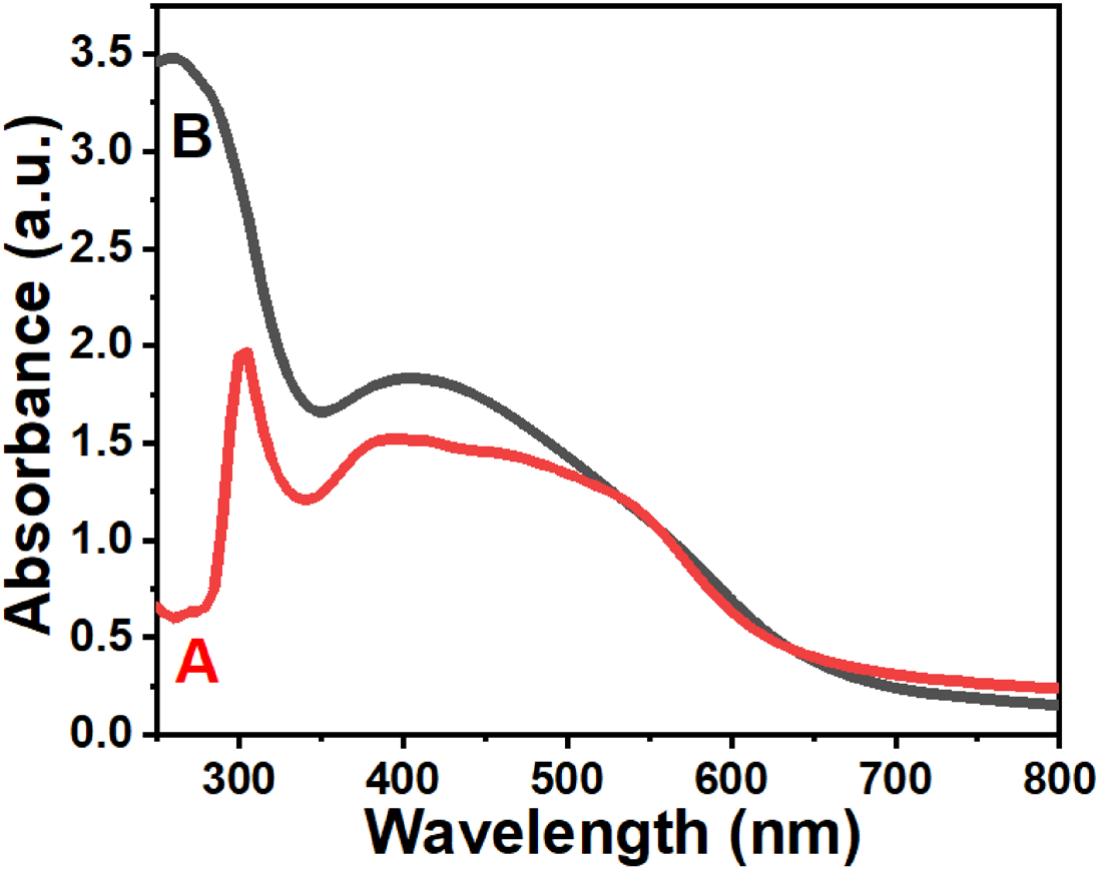
UV–Vis spectra of (**A**) intracellular and (**B**) extracellular Ag NPs.

The change in colour of both prepared samples to a dark reddish-brown color is a preliminary sign of the reduction process of Ag^+^ into Ag. In Fig. 1, the intracellular Ag NPs spectrum A showed three unique peaks: a sharp peak in the UV region at 305 nm and two broad bands in the visible range at 395 and 450 nm. These peaks might be due to the capping nature of silver nanoparticles, which could exhibit surface plasmon resonance (SPR) at the three wavelengths stated, or they could be due to our synthesized nanoparticles being combined into a core–shell with biomolecules such as proteins^49,50^. The peak shoulder near 450 nm was referred to as the heterogeneous size distribution of the silver nanoparticles according to Patil and Chougale^51^. The extracellular Ag NPs in Fig. 1’s spectrum B revealed their typical peaks: a sharp band in the UV at 260 nm and a wide band in the visible range at 405 nm. The blue shift of the absorption spectrum and surface plasmon resonance (SPR) of the extracellular Ag NPs to lower wavelengths relative to the intracellular Ag NPs may be ascribed to the decrease in the particle size of Ag NP^52^. The shape and size of Ag NPs may influence the position of the absorption peak and the number of bands^51^. One can see the asymmetrical shapes of the broad bands in the visible light range for both samples, which is more pronounced in the case of intracellular Ag NPs than the extracellular Ag NPs. This may be ascribed to the shoulders of the SPR peaks towards 550 nm being from the aggregations of the intracellular nanoparticles. Also, extracellular Ag NPs had a higher primary absorption peak intensity than intracellular Ag NPs, implying a larger population of monodisperse nanoparticles and slower aggregation formation for extracellular Ag NPs due to their loaded on the surface of biological polygonal structures from *B. cereus* cells, as we discussed in the next sections.

### Function groups of intracellular and extracellular Ag NPs

**Figure 2:**
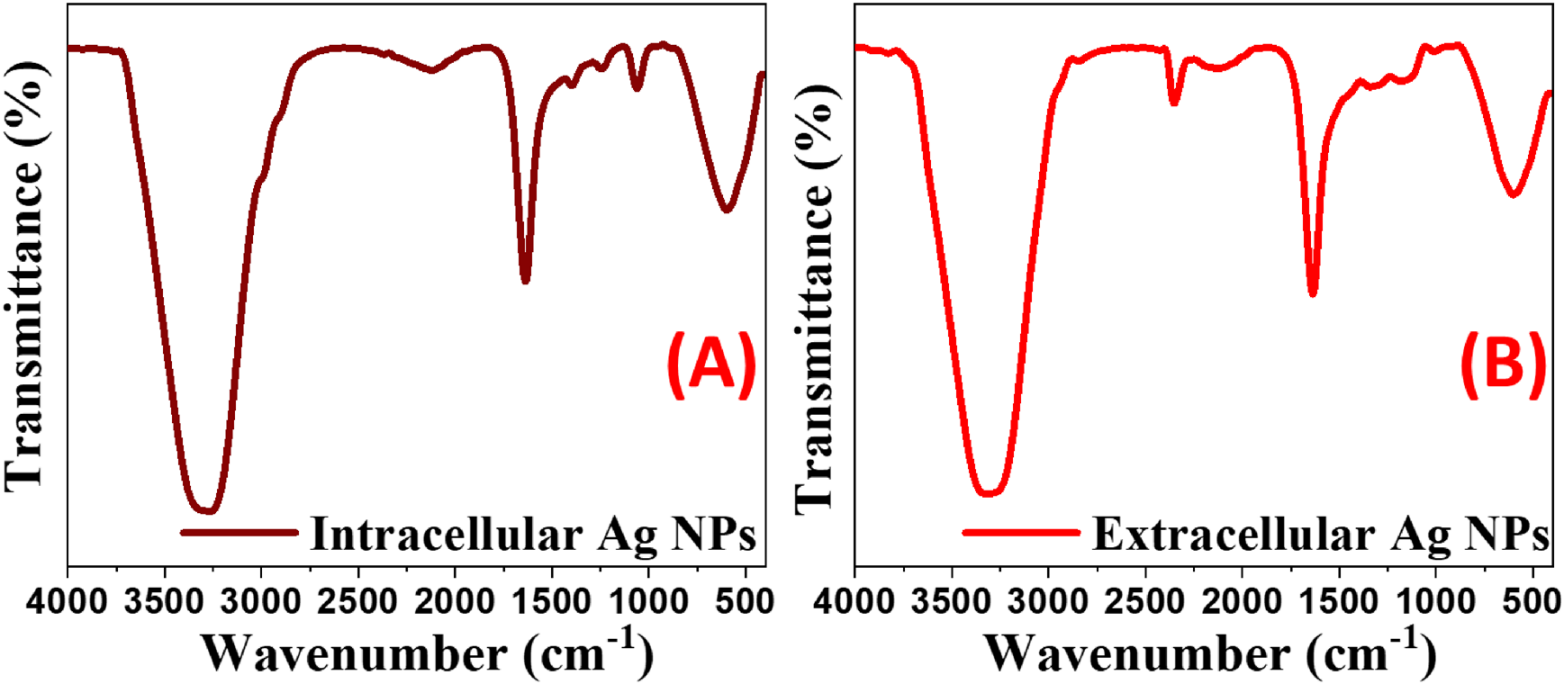
FTIR spectra of intracellular (**A**) and extracellular (**B**) Ag NPs.

Figure 2 shows the FTIR spectra of biosynthesized intracellular and extracellular Ag NPs, which were measured with a resolution of 4 cm^–1^ from 4000 to 400 cm^–1^. It illustrated how functional groups were integrated into biosynthesis through two reduction and stabilization steps. The reduction step involves employing reducing agents to convert Ag^+^ to Ag° atoms, and the stabilization phase involves capping the produced Ag with biochemicals that act as stabilizing agents^53^. Ibrahim et al. used the FTIR investigations to find out the connections between silver and bioactive materials that are responsible for the creation and constancy of nanoparticles as capping agents^13^. The FTIR spectrum of biosynthesized Intracellular silver nanoparticles with *B. cereus* cells, Fig. 2A, showed different peaks at 3296, 2124, 1635, 1390, 1236, 1069, and 601 cm^–1^. The strong peak at 3296 cm^–1^ revealed the hydroxyl (O–H) group of carboxylic acid or it may reveal the (N–H) group of the amides^32^. Moreover, the absorption band at 2124 cm^–1^ was for the C=C group of alkenes and the peak at 1635 cm^–1^ was attributed to the carbonyl (C=O) group stretching. These findings may reveal the presence of proteins that could reduce and stabilize the silver nanoparticles. Similarly, the moderate band at 1390 cm^–1^ represented the presence of the CH_3_ group bending or NO_2_ group according to Wagi and Ahmed^54^. The apparent bands at 1236 and 1069 cm^–1^ might reveal the stretching of the (C–O–C) group. The broadband at 601 cm^–1^ was attributed to (C–H) bonding out of plane bending vibrations^55^. The FTIR spectrum of extracellular silver nanoparticles that are biosynthesized by *B*. *cereus* cells showed different peaks at 3318, 2354, 2124, 1635, 1321, 1167, and 610 cm^–1^ in Fig. 2B. The sharp band at 3318 cm^–1^ was also attributed to O–H stretching in the prepared sample which may be due to the presence of hydroxyl-containing compounds like carboxylic acid, phenols, or alcohols. According to Badmus et al., the strong bands at 3296 and 1636.5 cm can be attributed to (O–H) hydroxyl groups and some free amide I (N–H) bending of biochemicals present in extracellular Ag NPs^56^. The appearance of the carbonyl group (C–O) vibrational stretching band at 2354 cm^–1^ could confirm the presence of other containing carbonyl compounds like carboxylic acids, amino acids, amides, or saccharides that are responsible for the reduction of Ag^+^ ions^25^. The peak at 2124 cm^‒1^ may reveal the C≡C bond stretching of alkyne^16,57^. The corporation of the free OH or negatively charged oxygen of the carboxylic group of amino acids that were present in our manufactured samples was visible in the IR fingerprint between 400 and 1500 cm^–1^. The discrepancy in fingerprint band locations between the two samples might be attributable to the various substances utilized in the two samples. The observable bands at 1321, 1167, and 610 cm^‒1^ may reveal Ag‒O, Ag‒N, or Ag‒C bonding that may contribute to the stabilization of the silver nanoparticles. According to Ibrahim and his coworkers, the links between nanoparticles and proteins like enzymes are the unbound amine groups of nanoparticles and the negative carboxyl groups of enzymes^13^. These proteins or enzymes could be released from the bacterial cell. They were responsible for the translation and stabilization of silver nanoparticles^13^. Prakash et al. highlighted earlier research suggesting that proteins play a role (function) in the nanoparticles binding via free amine or cysteine residues in amino acids^58^. According to Vigneshwaran et al., negative charges on carboxylate groups in existing enzymes in the bacterium's cell wall incorporate an electrostatic attraction towards these nanoparticles, causing their nature to be stable by these protein^59^. In conclusion, previous studies and our findings show that proteins can bind to nanoparticles via free amine groups or cysteine residues in proteins, or by the electrostatic attraction of negatively charged carboxylate groups in enzymes located within or outside *B. cereus* cells^13,16,23,39,60^.

### Morphological properties of intracellular and extracellular Ag NPs

**Figure 3:**
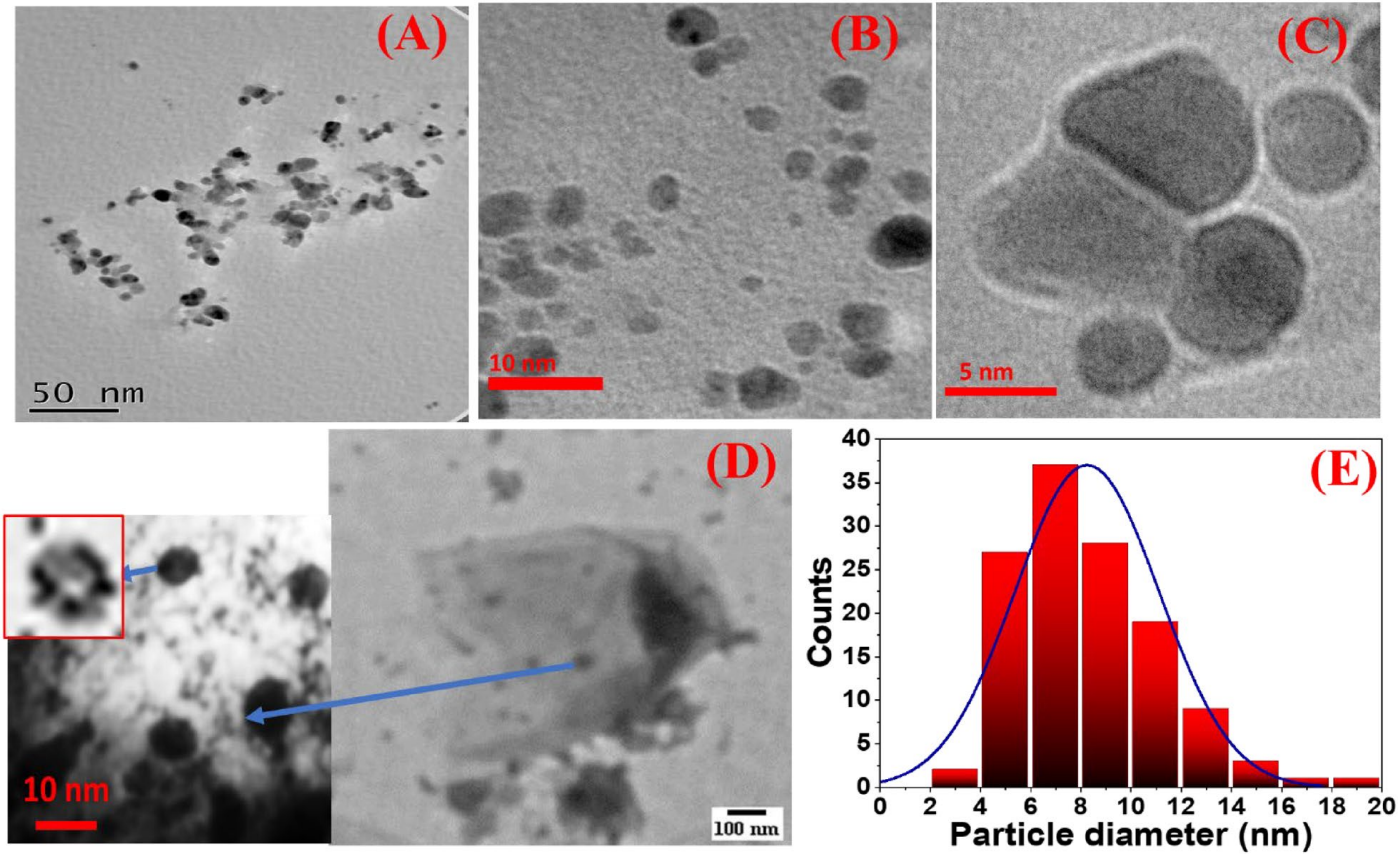
(**A**–**C**) TEM images of the intracellular Ag NPs at different magnifications, (**D**) TEM image showed damage in a bacterial cell that can give more information about the intracellular synthesis, and (**E**) histogram of the nanoparticle size distribution for the intracellular Ag NPs.

**Figure 4:**
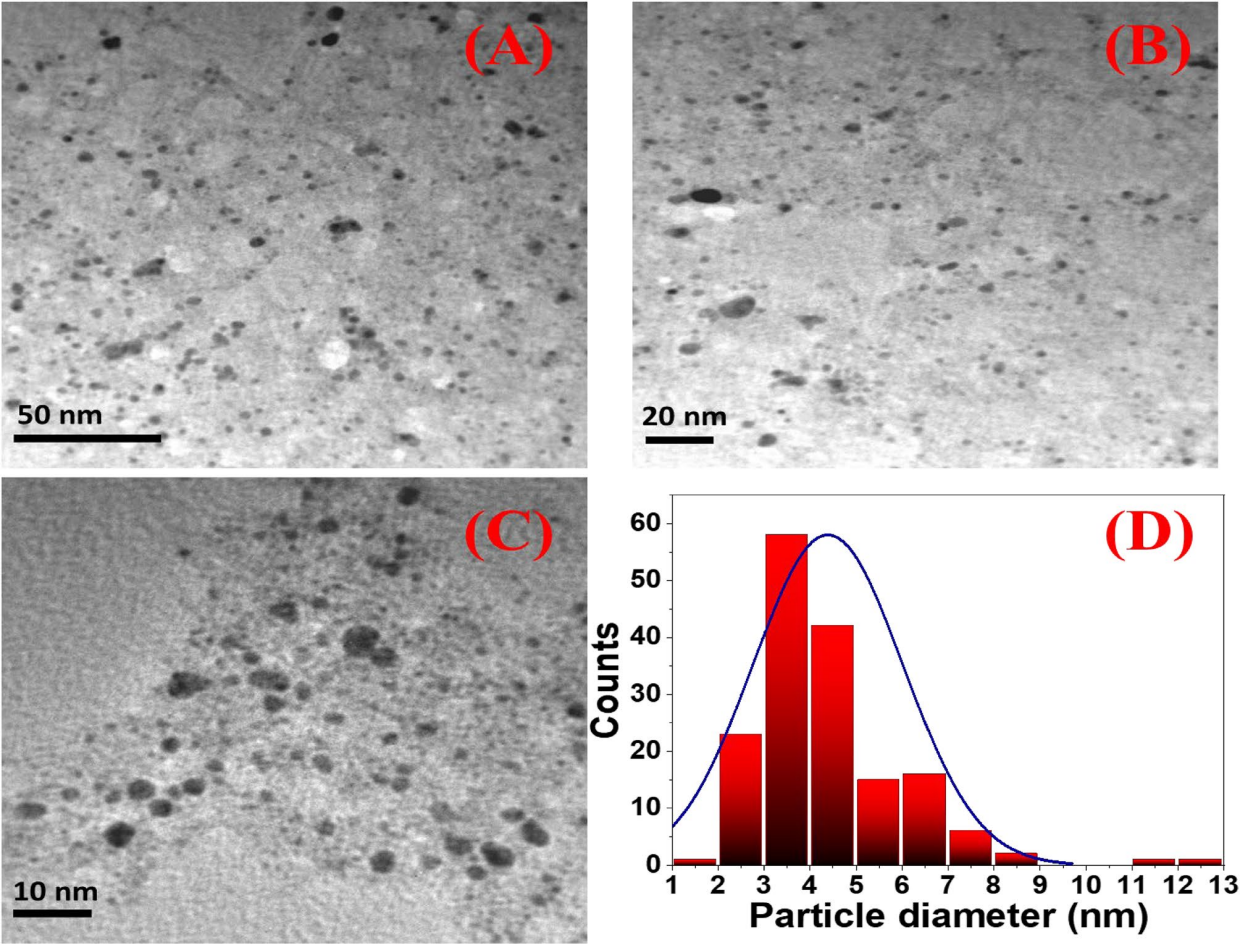
(**A**–**C**) TEM images of the spherical extracellular Ag NPs at different magnifications, and (**D**) histogram of the particle size distribution for the extracellular Ag NPs.

**Figure 5:**
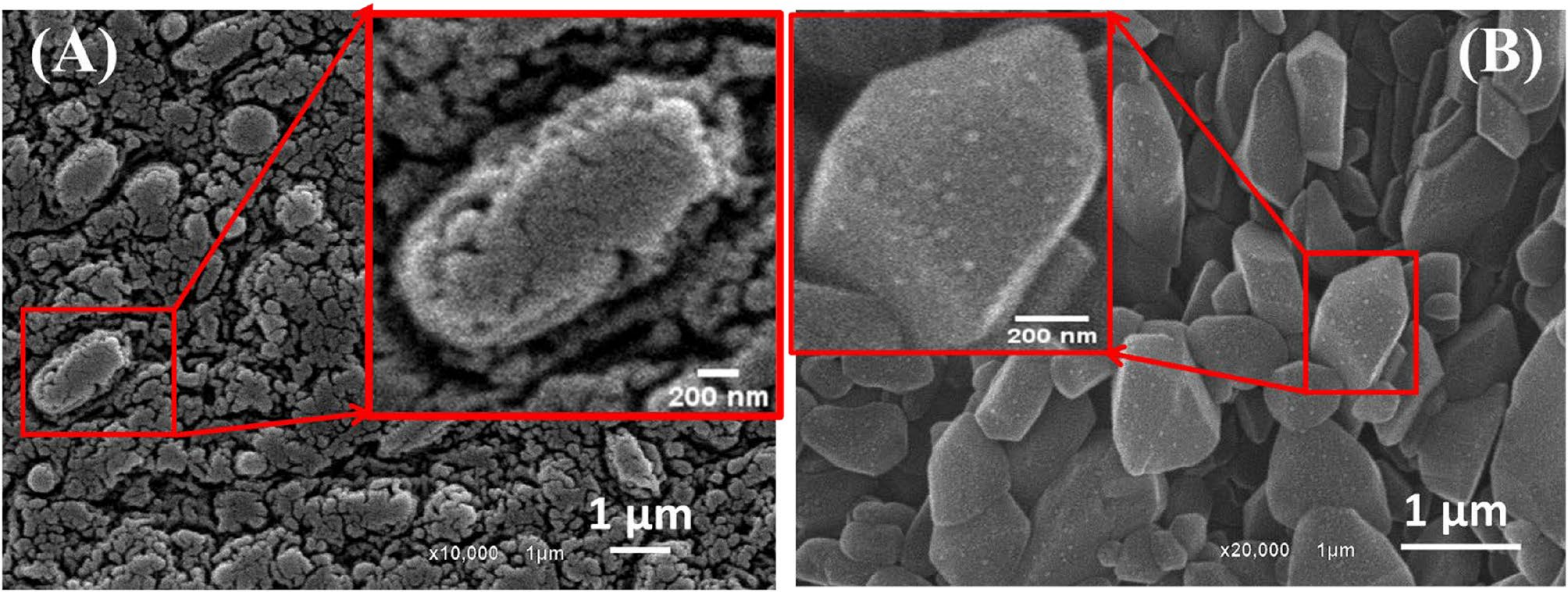
SEM images of (**A**) intracellular Ag NPs and (**B**) extracellular Ag NPs.

The distinct morphological features of the intracellular and extracellular Ag NPs were characterized in Figs. 3, 4, and 5 utilizing HR–TEM and SEM images. The observed Ag NPs were spherical and ellipsoidal as mentioned in the case of intracellular silver nanoparticles in Fig. 3A–C. These spherical nanoparticles were synthesized inside the *B. cereus* cells which are imaged at low magnification as shown in Fig. 3D. The TEM investigation of *B. cereus* revealed significant structural damage at the cellular level and irreversible cell membrane rupture at many locations with the apparent leakage of intracellular contents. These TEM images were utilized to calculate the average particle diameter distribution for the intracellular Ag NPs. It was found in Fig. 3E that the diameter of the intracellular Ag NPs is ranged from 3.7 to 18.9 nm with an average of 8.2 nm. The extracellular Ag NPs were examined in Fig. 4A–C and characterized as separated spherical nanoparticles and homogenous in shape with no direct contact as synthesized by Hoang et al.^61^. According to our TEM image findings, the average diameter of the Ag NPs was 4.01 nm and ranged from 1.8 nm to 12.4 nm in Fig. 4D, indicating that the Ag NPs included quantum dots. As proven by Le Nhat Trang et al., the smaller size can boost the electroactive surface area, electron-transfer kinetics, and electrode stability^62^. Their research found that biosynthesized Ag NPs made from three different plant extracts had remarkable size-dependent catalytic characteristics in the electrochemical detection of 4-nitrophenol in food samples. The morphological structures of intracellular and extracellular Ag NPs were observed using a scanning electron microscope (SEM) image as shown in Fig. 5. Figure 5A displays spherical nanoparticles for intracellular Ag NPs, whereas Fig. 5B reveals regular morphologies of polygonal structures for extracellular Ag NPs for the first time. The magnified SEM image in Fig. 5A showed the Ag NPs that covered the membranes of the bacterial cells. The inset magnified SEM image of Fig. 5B showed densely distributed Ag NPs on the surface of polygonal structures.

Our suggested process for the production of Ag NPs has two steps: reduction and capping or stabilization. According to Mohamed et al., the *B. cereus* bacterial cells died following treatment of the bacterial pellet with an aqueous solution of Ag^+^ ions because the silver ion precursor, silver nitrate, is an antibacterial agent^63^. Because of the electrostatic affinity between positively charged Ag^+^ ions and negatively charged carboxylate groups on the bacterial membrane, Ag^+^ ions are often harmful to bacterial cells. This is supported by the lack of *B. cereus* growth in the created Ag nanoparticles, as well as the absence of biofilm formation following Ag NP synthesis. We found that *B. cereus* cells could not survive after these nanoparticles were synthesized. The reduction process required biochemicals such as the nitrate reductase enzyme produced inside *B. cereus* cells, and the capping process required biochemicals such as the peptidoglycan polymer present on the gram–positive bacterial cell wall to provide stability to the Ag nanoparticles. The bacterial cell wall also protects and preserves the structural integrity of the bacteria's cellular components^64^. The structural unit of peptidoglycan polymer is composed of glycan strands consisting of repeating disaccharides of NAG and NAM-pentapeptide^64^. The cell wall thickness of gram–positive bacteria such as *B. cereus* ranges from 20 to 80 nm^65,66^. This explains the thick capping layer of intracellular Ag NPs, which are produced inside the *B. cereus* cell and then processed for capping by incorporating a portion of the cell wall. This is further corroborated by the presence of carbohydrate functional groups as observed in the FTIR data, Fig. 2A. According to Pandian et al., the high concentrations of silver nitrate showed different effects on bacteria^67^. The killing of bacteria is done by different mechanisms. One of them was binding to the thiol groups of protein and denaturing them. The second mechanism was programmed cell death, the bacterial cell undergoes apoptosis. The third one is causing the DNA to be in the condensed form, not in the relaxed form, which inhibits cell replication. While at low concentrations, bacteria try to survive in the new environmental conditions and the synthesis of silver nanoparticles may be occurred. After the synthesis of the Ag NPs, the *B. cereus* cannot survive in the presence of the Ag NPs due to the potential antibacterial action of the Ag NPs. Recent cases reported by Mohammed et al. also support similar hypothesis^63^.

The spherical Ag nanoparticles (Fig. 5A) were synthesized internally but accumulated on the exterior wall of B. cereus based on the following mechanism. The silver ion was attached to the surface of the cell wall of the *B. cereus* and reduced through the C=O group of aldehydic, ketonic polysaccharides or amino acids that appeared at 1634.5 cm^–1^ and 1642 cm^–1^ as observed in the FTIR spectra, Fig. 2. Our interpretation is the first time to confirm this suppose.

The extracellular production of Ag nanoparticles using *B. cereus* is now extensively known. The shapes of the extracellular Ag nanoparticles were spherical in origin, according to the assessment of their shape and size. The spherical Ag NPs can be seen on the surface of the polygonal structures of the magnified SEM image in Fig. 5B as extremely small and bright spots. They may also be capped based on the visible peaks in the UV (Fig. 1B) and FTIR spectrum (Fig. 2B). Logically, the capping process prevents agglomeration. But here the produced Ag NPs outside the *B. cereus* cells were loaded on the surface of these polygonal structures. It may demonstrate that the polygonal structures were biological compounds produced from *B. cereus* cells and the reduction of Ag^+^ occurred on their surfaces. The reduction process is like the attachment of the metallic ions to electron–donating groups of these bioorganic compounds. The reduction process produces stable Ag NPs on the surface of the polygonal structures. These Ag NPs were only removed by ultrasonication of a diluted solution of extracellular Ag NPs for a long time for the TEM investigation in Fig. 4A–C, and they appeared as extremely small spherical nanoparticles.

### Structural properties of intracellular and extracellular Ag NPs

**Figure 6:**
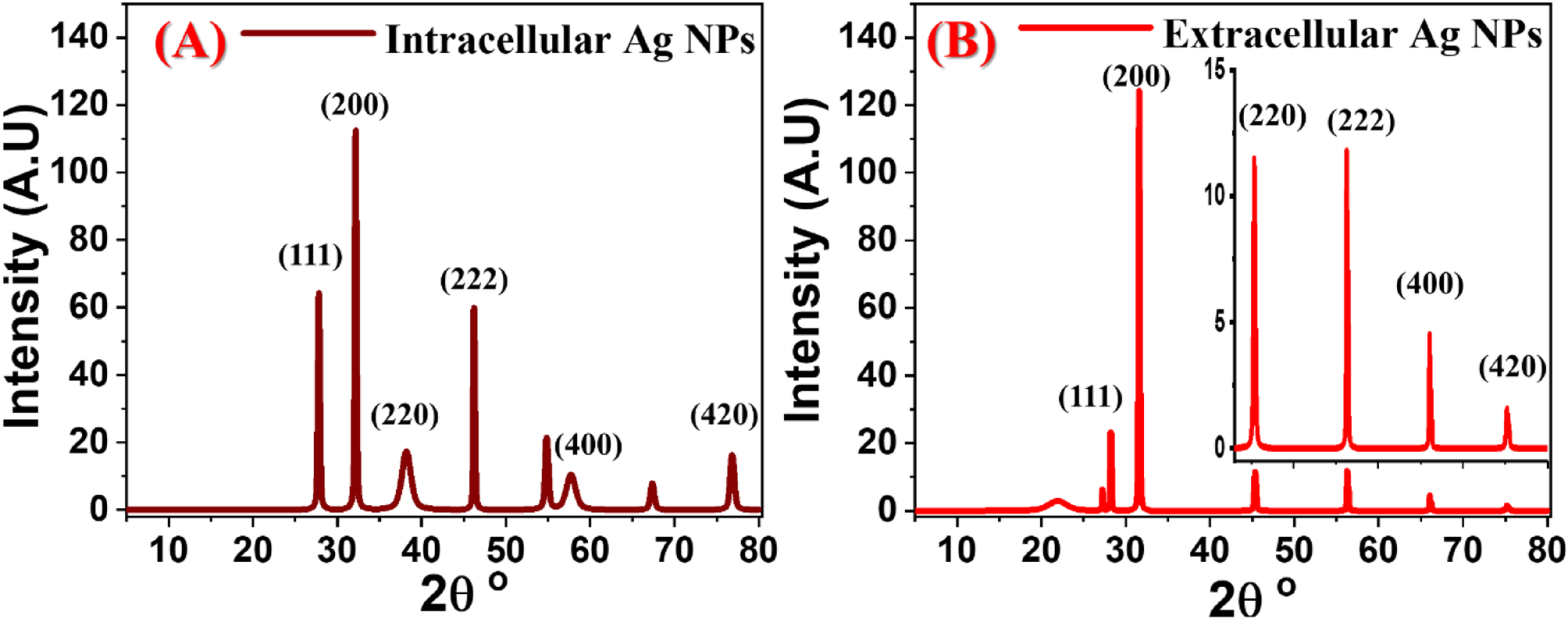
XRD patterns of the (**A**) intracellular and (**B**) extracellular biosynthesized Ag NPs.

Figure 6 illustrated XRD patterns of intracellular and extracellular biosynthesized silver NPs. They were coated on glass slides to develop the XRD analyses of the two prepared samples. Silver nanoparticles’ crystalline nature is evaluated by recording the XRD from 5° to 80°. The characteristic XRD peaks of the intracellular Ag NPs, Fig. 6A, revealed their 2θ° positions at 27.50°, 32.11°, 38.09°, 46.11°, 54.86°, 57.73°, 67.37°, 76.85°. On the other hand, the peaks in Fig. 6B were positioned at 27.05°, 28.20°, 31.65°, 45.44°, 56.26°, 66.14°, and 75.35° belonging to the extracellular Ag NPs. The most important characteristic peaks in both spectra are correlated to the indexing Miller indices (*hkl*) values as shown in Fig. 6. They were indexed to the standard planes of crystalline Ag NPs with FCC structure according to the Joint Committee on Powder Diffraction Standards (JCPDS) card No. 04-0783^17,22,61,68^. The (200) is the preferred orientation for the growth of Ag NPs. Other peaks may be associated with the bioorganic molecules employed in the biosynthesis of Ag NPs. Prakash et al. used *B. cereus* to get an XRD pattern that revealed the crystalline structure of silver NPs with (111), (200), and (220) planes^58^. The crystallite size of the intracellular and extracellular biosynthesized Ag NPs are calculated using Scherrer’s Eq. (1)^69,70^.

1 $$D=\frac{0.95 \lambda }{{\beta }_{o}\mathrm{cos}\theta }$$

where, ${\beta }_{o}$ is the full width at half maximum (FWHM) of the peak, $\lambda$ is (0.154178 nm) the wavelength of incident radiation and $\theta$ is Bragg’s angle. The FWHM value was used to calculate the average size of the nanoparticles. The average crystallite sizes of the intracellular and extracellular biosynthesized Ag NPs are 45.4 nm and 90.8 nm, respectively.

### Hydrodynamic size and zeta potential of intracellular and extracellular Ag NPs

**Figure 7:**
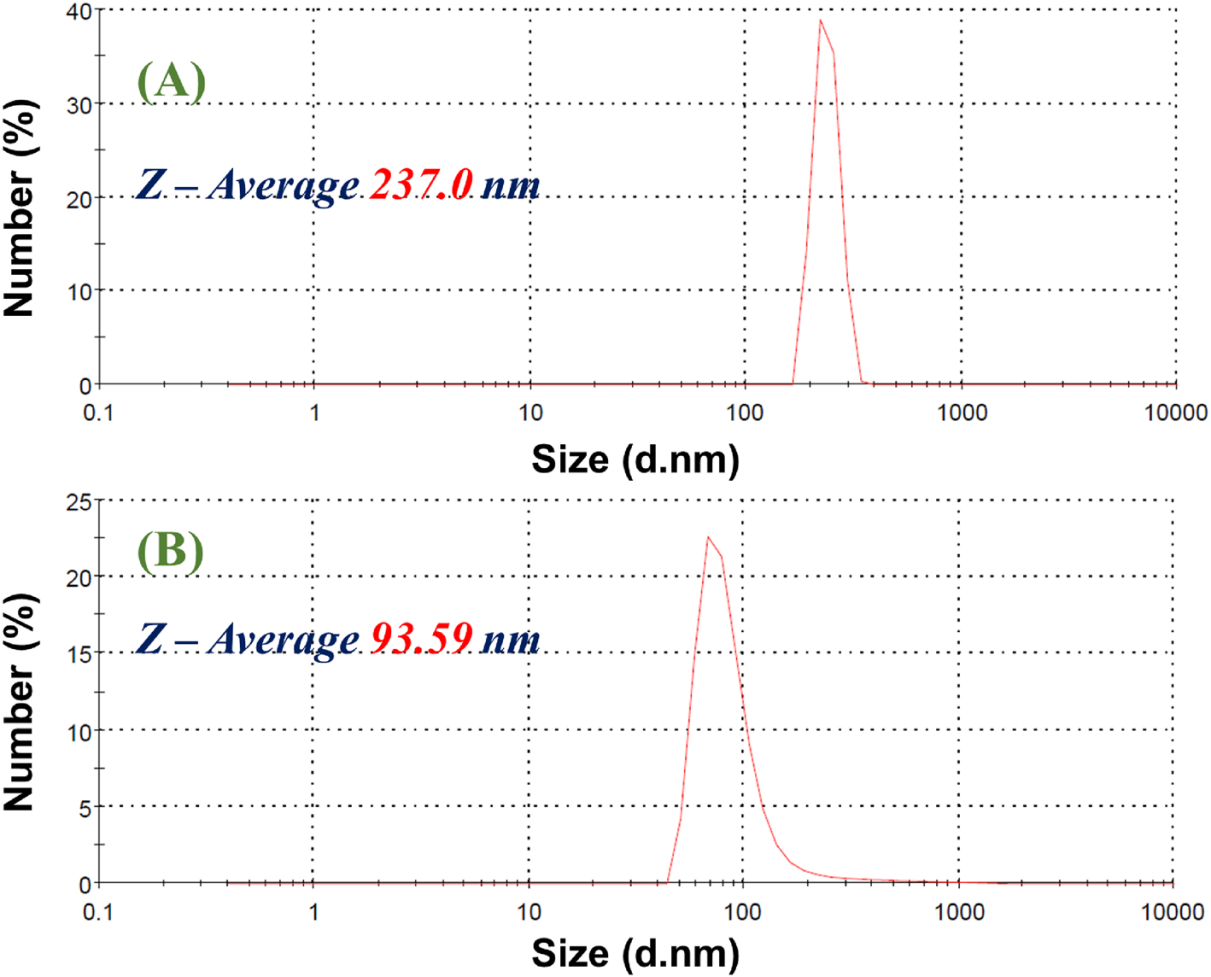
DLS patterns of (**A**) intracellular and (**B**) extracellular biosynthesized Ag NPs.

Figure 7A and B shows the hydrodynamic size of intracellular and extracellular Ag NPs, respectively, using the DLS (Dynamic Light Scattering) investigation. The size of the intracellular Ag NPs was 237.0 nm, whereas the size of the extracellular Ag NPs was 93.59 nm. Hence, the extracellular Ag NPs were smaller in size than the intracellular Ag NPs, which agrees with the behaviour observed by SEM and TEM measurements. However, the sizes observed by DLS are higher than the values observed by the TEM analysis. The influence of Brownian motion causes higher size in the case of DLS than TEM.

The zeta potential was also measured for both samples. Intracellular Ag NPs had a zeta potential of – 4.71 mV, while extracellular Ag NPs had a zeta potential of – 20.22 mV. Hence, the zeta potential of our capped Ag NPs was negative, indicating that the used reducing agent had negatively charged functional groups that contribute to the Ag NPs’ colloidal stability. Also, the surface of extracellular Ag NPs is more stable because it has more negative electrical charges than the intracellular Ag NPs. The higher negative zeta potential denotes higher stability and better colloidal properties due to electrostatic repulsion, and the higher dispersity^51^.

### The catalytic activity of the intracellular and extracellular Ag NPs

**Figure 8:**
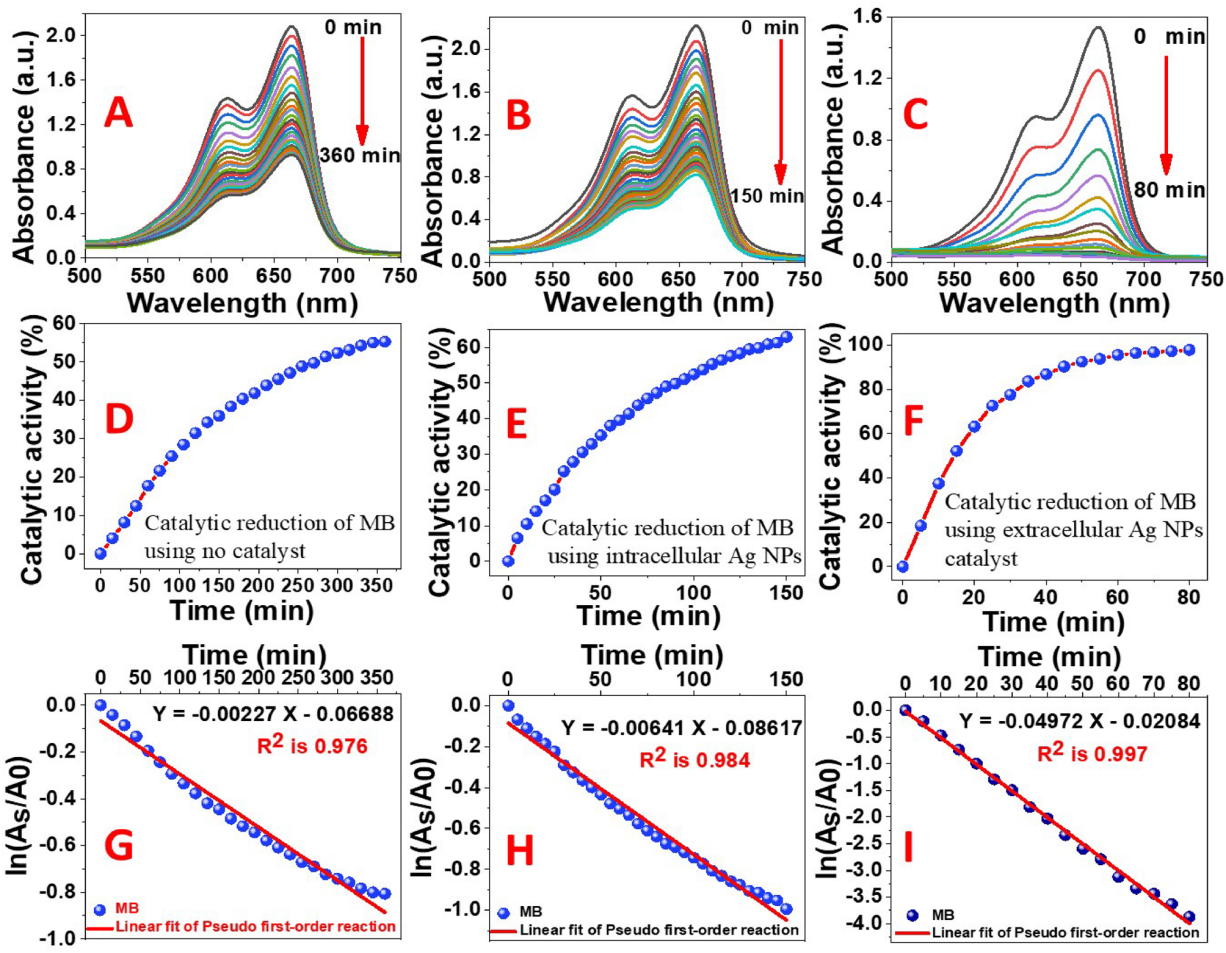
Absorption spectra of MB in an aqueous medium at different time intervals in the (**A**) absence of Ag NPs catalyst and the presence of (**B**) intracellular and (**C**) extracellular Ag NPs; their catalytic reduction percentages (**D**–**F**); and their first order kinetic modelling (**G**–**I**), respectively.

**Scheme 1:**
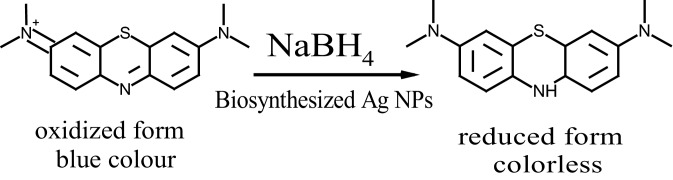
Catalytic reduction of MB using NaBH_4_ as a reducing agent and the biosynthesized Ag NPs as a catalyst.

5 mL of 0.1 M NaBH_4_ was used to examine the catalytic reduction of 25 mL of highly concentrated MB (50 ppm) by 100 μL of as–synthesized Ag NPs solutions. The UV–Vis spectra were also recorded with sequential time intervals for MB/Ag NPs solutions incubated in dark conditions to prevent photocatalytic reactions. A potential application of Ag NPs catalytic activity was the reduction of aqueous MB to Leuco–MB in the presence of excess NaBH_4_. At room temperature, the reaction was monitored with a UV–Vis spectrophotometer in the wavelength range from 500 to 750 nm. The maximum absorption peak of MB in an aqueous medium was positioned at 665 nm^71^. According to Reddy et al., the reduction of MB by NaBH_4_ in the absence of nanocatalyst was measured for 120 min with a modest decreasing trend in the maximum absorbance, indicating that MB was reduced at a very slow rate^72^. The UV–Vis spectra of the reduction of MB by NaBH_4_ in the absence and presence of catalytically active Ag NPs are shown in Fig. 8A–C. The reduction of 50 ppm MB in the absence of catalysts was assessed in our study, and the control test took 6 h and reached ~ 55%@360 min, Fig. 8D. The reduction process was found to be accelerated in the presence of our catalysts colloids which showed a rapid reduction in the absorption intensity of MB solution in the case of the intracellular and extracellular Ag NPs, Fig. 8B and C. It was previously reported that Ag NPs help in the electron relay from BH_4_^–^ (donor) to MB (acceptor). BH_4_^–^ ions are nucleophilic, while MB (cationic dye) are electrophilic in nature with respect to Ag NPs. Therefore, the Ag NPs accept electrons from BH_4_^–^ ions and deliver them to the MB, as shown in Scheme 1^73^. The absorption peak at 665 nm for MB dye was found to decrease rapidly in the case of extracellular Ag NPs with an increase in the reaction time to 80 min. The spectra in Fig. 8B and C indicate that the dye has been degraded at a faster rate in the case of the extracellular than those of the intracellular. This might be because extracellular Ag NPs had a smaller diameter and were not as well capped as intracellular Ag NPs^72,74^.

The synthesized sample’s catalytic efficiency of MB reduction was calculated using Eq. (2). The rate constant of the reaction (*k*_*app*_) was determined from the linear plot between $\mathrm{ln}(\frac{{A}_{s}}{{A}_{o}})$ and time (min) according to the pseudo-first-order kinetics, Eq. (3):

2 $$\mathrm{Catalytic\, activity \%}=\frac{{A}_{o}-{A}_{s}}{{A}_{o}}\times 100$$

3 $$\mathrm{ln}\left(\frac{{A}_{s}}{{A}_{o}}\right)=-{k}_{app}t$$

where *A*_*s*_ and *A*_*o*_ are the absorbances of MB at zero (min) and time *t* (min), respectively. The control reaction was established for understanding the catalytic activities. The degradation efficiency was calculated using Eq. (2) and presented in Figs. 7D–F. The extracellular Ag NPs exhibited high catalytic degradation efficiency close to 100% after 80 min, Fig. 8F. Whereas the efficiency of MB reduction by the intracellular Ag NPs, Fig. 8E, was ~ 63% after 150 min versus ~ 55% after 360 min in the absence of Ag NPs. The linear correlation between $ln\left(\frac{{A}_{s}}{{A}_{o}}\right)$ versus reduction time in minutes, Fig. 8G, H, and I, indicates that the reduction follows a pseudo-first-order reaction kinetics. The rate constants were calculated from the slopes of these graphs and presented in Table 1.

**Table 1:** Data obtained from the catalytic MB reduction in the absence and presence of our biosynthesized intracellular and extracellular Ag NPs catalysts.

Catalyst	Reduction of MB (%)	Time (min)	*k*_*app*_ (min^–1^)	R^2^	$\frac{{({K}_{app})}_{{\text{with}} \, {\text{catalyst}}}}{{{(K}_{app})}_{{\text{without}} \, {\text{catalyst}}}}$
Extracellular Ag NPs	~ 98%	80 (min)	0.04972	0.997	~ 22
Intracellular Ag NPs	~ 63%	150 (min)	0.00641	0.984	~ 3
Distilled water	~ 55%	360 (min)	0.00227	0.976	Control

The catalytic activity of our extracellular Ag NPs was higher than that of our intracellular Ag NPs, as shown in Table 1. Using basic mathematical Eq. (4), the extracellular catalyst raised the rate of MB reduction by 22 times, whereas the intracellular catalyst increased the rate of MB reduction by 3 times.

4 $$\mathrm{The} \, \mathrm{rate} \, \mathrm{of} \,  \mathrm{increase }=\frac{{({K}_{app})}_{\mathrm{with} \,  \mathrm{catalyst}}}{{{(K}_{app})}_{\mathrm{without} \, \mathrm{ catalyst}}}$$

**Figure 9:**
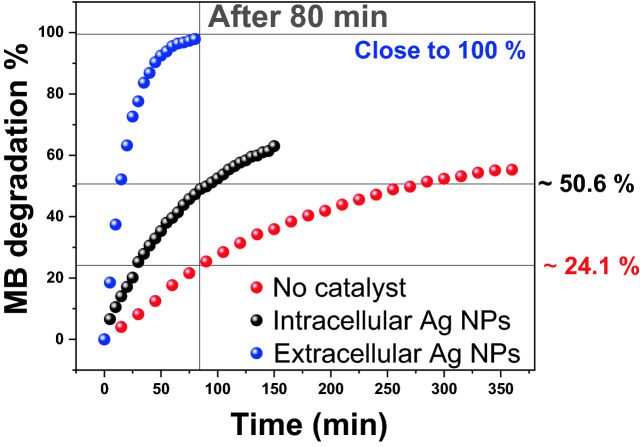
MB degradation efficiency at different time intervals in the absence and the presence of intracellular and extracellular Ag NPs.

These findings showed that biosynthesized extracellular Ag NPs samples had an exceptional capacity to efficiently remove high concentrations of hazardous dyes from contaminated water in less time, as shown in Fig. 9. Comparative plots of MB degradation % in aqueous solutions at different time intervals for the control reaction and in the presence of intracellular and extracellular Ag NPs are shown in this figure. The MB degrading efficiency reached 24.1%, 50.6%, and 100% after 80 min reduction time, respectively.

Extracellular Ag NPs greatly boosted the rate of MB reduction as compared to intracellular Ag NPs due to their small size, high surface area, and greater negative potential. Furthermore, E° (MB) =  + 0.01 V > E° (intracellular Ag NPs) =  − 0.00471 V > E° (extracellular Ag NPs) = − 0.02022 V > E° (BH_4_^−^) = − 0.21 V, which represents the ideal conditions for an effective relay of electrons between the acceptor (MB) and the donor (NaBH_4_). As a result, extracellular Ag NPs were the electrophiles that could receive electrons from BH_4_^–^ and transport them to MB faster than the other electrophile.

## Conclusion

*Bacillus cereus* has been employed for the first time in one-pot biosynthesis of capsulated Ag NPs, employing both intracellular and extracellular approaches. To distinguish between the nanostructures created by these two processes, numerous techniques are used to examine their structures, morphologies, optical characteristics, hydrodynamic diameters, and zeta potentials. The bioactive components responsible for the reduction of Ag^+^ ions into Ag and the formation of stable encapsulated Ag NPs were identified using FTIR spectroscopy. Spherical and polygon nanomorphology of a face-centred cubic structure with average crystallite diameters of 45.4 and 90.8 nm were reported for intracellular and extracellular biosynthesized Ag NPs, respectively. The catalytic activities of intracellular and extracellular biosynthesized Ag NPs were evaluated for the reduction of highly concentrated MB dye solution in terms of the reduction efficiency and reduction rate relative to the noncatalytic reduction test. It was found that the extracellular biosynthesized Ag NPs exhibited a strong capacity to degrade MB than intracellular Ag NPs. Extracellular Ag NPs reduced MB by 100% after 80 min, compared to 50.6% and 24.1%, respectively, in the presence and absence of intracellular Ag NPs. The extracellular catalyst increased the rate of MB reduction by 22 times, whereas the intracellular catalyst increased it by 3 times. As a result, the Bacillus cereus bacteria-based extracellular synthesis of Ag NPs might be used in industry as a cost-effective solution for eliminating the harmful MB dye from the industrial wastewater.

## Materials and methods

### Materials

All chemicals and media used were of analytical grade. *B. cereus* strain was kindly provided by RCMB, The Regional Centre for Mycology and Biotechnology, Al-Azhar University, Cairo, Egypt. The *B. cereus* bacteria were grown on nutrient agar at 37 °C for 24 h according to Murary et al.^75^. The pure culture was stored on nutrient agar (Himedia, India) slants at 4 °C. Nutrient broth (NB) was also purchased from (Hi-media, India) for *B. cereus* biomass cultivation. Silver nitrate (AgNO_3_) was purchased from TITAN BIOTECH with a ≥ 99.5% purity. Sterile deionized water was used throughout the experiment.

### Methods

#### Preparation of *B. cereus* strain culture

To prepare bacterial culture for biosynthesis studies, *B. cereus* was grown aerobically in 100 mL nutrient broth in a 250 mL Erlenmeyer flask. The flasks were inoculated, incubated for 120 h orbital shaker at 150 rpm, and maintained at 28 ± 2 °C. The harvested bacterial culture, the bacterial pellet, was collected at 4000 rpm and 4 °C, using a cooling centrifuge. After the development period, the culture supernatant was filtered to obtain a pure solution. The collected bacterial cells, the precipitates, were washed using sterile deionized water. Herein, we have the bacterial pellet and the filtrate for the intracellular and extracellular bio-capped Ag NPs, respectively.

#### Preparation of intracellular and extracellular bio-capped Ag NPs

All bacterial pellet and the filtrate, separately, were directly introduced into Ag NPs synthesis as the reducing and the stabilizing agents under normal laboratory room temperature and pressure (NTP) conditions. Approximately, 0.1 g of the bacterial pellet was added into 100 mL of 1 mM AgNO_3_ aqueous solution for intracellular biosynthesis. The filtrate, 100 mL of it, reacts with 100 mL of the same aqueous solution for extracellular biosynthesis. The colour of both reactions was observed to be changed instantaneously into reddish-brown which is evidence of the formation of Ag NPs which are monitored by UV–Vis spectrophotometer. In consideration, the negative controls were used in this study.

#### Characterization of the intracellular and extracellular BC-capped Ag NPs

The optical spectra of the prepared intracellular and extracellular BC-capped Ag NPs were recorded by a UV–Vis double-beam spectrophotometer (λ-950, Perkin Elmer, USA). FTIR spectroscopy (8400 S Shimadzu, UK) was used to determine the functional groups of chemical components for both. The intracellular and extracellular BC-capped Ag NPs’ morphologies and size were characterized by a high-resolution TEM with an accelerating voltage of 200 kV (HRTEM, JEOL-2010F, JEOL, Japan) and SEM with accelerating voltage ranges from 0.5 to 30 kV (JSM–6510, JEOL, Japan). The particle diameter distribution histograms were determined using HRTEM images of both intracellular and extracellular Ag NPs and using image-J software^76^. XRD charts of the intracellular and extracellular BC-capped Ag NPs were measured by X-Ray diffractometer (XRD, Philips X’Pert Pro MRD with CuK_α_ line of wavelength 0.154 nm), operated at 40 kV along with 20 mA in 2θ range from 10° to 80° with a 2° min^–1^ scanning velocity. DLS and zeta potential studies were also performed for the prepared samples using (Zetasizer Nano ZN, Malvern Panalytical Ltd, UK) at a pH of 7 and a temperature of 25 °C.

#### The catalytic activity of the intracellular and extracellular Ag NPs

The catalytic activity study of the biosynthesized samples was performed at room temperature (25 ± 2 °C) using the reaction of methylene blue dye with sodium borohydride in dark conditions, according to Kordy et al. with some modifications^77^. The intracellular and extracellular Ag NPs' catalytic efficiencies were investigated and compared. This comparison was made up by using 25 mL of MB, 50 ppm, and using 5 mL of 0.1 M of sodium borohydride. This type of degradation is very slow in case of no catalyst is used. Using the same amount of catalysts makes the comparison between them better. The absorbance was monitored on a UV/visible spectrophotometer (Perkin Elmer 950-lambda, USA) at 665 nm and scanned from 500 to 750 nm. The catalytic activities were determined using Eq. (2) and the rate constant of the reaction was determined from the plot between $\mathrm{ln}(\frac{{A}_{s}}{{A}_{o}})$ and time (min) using Eq. (3). The control reaction was established for understanding the catalytic activities.

### Statistical tools

The analytical determinations were performed in triplicate. The results were expressed as the mean ± standard deviation (SD) according to Quirk and Rhiney^78^. The statistical significance was simply assessed by excel (Microsoft Office 365, US) at a significance threshold value of p < 0.05.

## Data Availability

The datasets used and/ or analysed during the current study available from the corresponding author on reasonable request.
